# Development and optimization of host DNA depletion in blood cultures using a saponin and salt-activated nuclease-based method

**DOI:** 10.3389/fmicb.2026.1784408

**Published:** 2026-04-08

**Authors:** Jawad Ali, Anurag Basavaraj Bellankimath, Jonathan Hira, Crystal Chapagain, Silje Therese Opgård, Gunnar Skov Simonsen, Rafi Ahmad

**Affiliations:** 1 Department of Biotechnology, University of Inland Norway, Hamar, Norway; 2 Department of Microbiology and Infection Control, University Hospital of North Norway, Tromsø, Norway; 3 Department of Medical Biology, Faculty of Health Sciences, UiT The Arctic University of Norway, Tromsø, Norway; 4 Faculty of Health Sciences, Institute of Clinical Medicine, UiT The Arctic University of Norway, Tromsø, Norway

**Keywords:** bloodstream infection and sepsis, host DNA depletion, nanopore sequencing, qPCR, SAN nucleases, saponin

## Abstract

Early diagnosis of sepsis and bloodstream infections is essential to reduce mortality and combat antimicrobial resistance. However, current diagnostic methods are slow and laborious. Nanopore sequencing has the potential to be a rapid diagnostic tool for sepsis. Nonetheless, extracting bacterial DNA from blood samples remains challenging due to the abundance of host DNA and the presence of inhibitory substances. This study aimed to develop and optimize a method using saponin and salt-activated nucleases (SAN) for efficient host DNA depletion in blood cultures. Different saponin concentrations (2%–5%), HL-SAN and M-SAN nucleases, and various salt conditions (NaCl and MgCl_2_) were tested for their ability to reduce host DNA in *Escherichia coli* and *Staphylococcus aureus* blood cultures. The effects of bead-beating durations of 6 and 10 min on DNA fragment size distribution were also evaluated. The optimized method was validated using spiked and clinical blood cultures of the most prevalent BSI causing pathogens. Results showed that 4% of saponin treatment combined with 250 units (10 μL) of SAN nucleases achieved efficient host DNA depletion in spiked blood cultures. Compared to undepleted samples, HL-SAN achieved up to 38-fold reduction in host DNA for both *E. coli* and *S. aureus*. Likewise, M-SAN resulted in ~10-fold reduction in *E. coli* and ~4-fold reduction in S. aureus samples. Although SAN enzymes combined with higher salt concentrations showed slightly better host DNA removal, the difference was not statistically significant. Shortening the bead-beating time to 6 min improved the recovery of longer DNA fragments compared to 10 min. The optimized method validated using spiked and clinical-positive blood cultures, demonstrated efficient host depletion and bacterial DNA recovery.

## Introduction

Bloodstream infections (BSIs) and sepsis are major health problems accounting for millions of deaths each year around the world (Fleischmann et al., 2016). Early and appropriate diagnosis of sepsis is critical for the patient’s survival. However, the current sepsis diagnosis is based on traditional blood culture, followed by biochemical methods, matrix-assisted laser desorption/ionization time-of-flight mass spectrometry (MALDI-TOF MS), polymerase chain reaction (PCR), and usually automated antibiotic susceptibility testing (AST) (Duncan et al., 2021). These methods, regarded as the gold standard for sepsis diagnosis, are time-consuming and can take over 72 h to identify pathogens and determine antibiotic resistance (Duncan et al., 2021). The delay is undesirable for septic patients, who require prompt antibiotic administration. Therefore, there is an urgent need to develop rapid diagnostic methods for BSIs and sepsis.

Multiplex PCR systems on positive blood cultures can identify the most common species and their main resistance genes in less than 60 h in most cases (Chen et al., 2024). However, multiplex PCR panels are limited to identifying only certain specific species and resistance markers and require regular updates to include new resistance patterns (Yang and Rothman, 2004; Payne et al., 2018; Caméléna et al., 2022; Enne et al., 2022). Metagenomic next-generation sequencing (mNGS) using nanopore technology (Oxford Nanopore, UK) is a promising method for rapid infection diagnosis. mNGS overcomes the limitations of multiplex PCR by detecting any pathogen or resistance markers in the sample and not relying on predefined targets (Chiu and Miller, 2019; Gu et al., 2019). Nanopore sequencing provides real-time data, low cost per sample, and the potential to be used directly on samples without culturing (Chiu and Miller, 2019; Hu et al., 2021). Several studies have demonstrated the potential of nanopore sequencing for detecting BSI-causing pathogens and their antibiotic resistance genes (ARGs) (Han et al., 2024; Harris et al., 2024; Govender et al., 2025; Nielsen et al., 2025). Nanopore sequencing of positive blood cultures has been shown to identify bacterial species with 97% sensitivity and 94% specificity in 3.5 h, compared to routine diagnostic methods (Govender et al., 2025). Additionally, antimicrobial resistance (AMR) results for the top 10 sepsis-causing pathogens were obtained 20 h earlier than routine AST (Govender et al., 2025). Similarly, Harris et al. (2024) demonstrated that nanopore sequencing of positive blood cultures achieved 94% concordance with routine diagnostic methods for species identification. A recent study has used nanopore-targeted sequencing, utilizing pathogen-specific primers for multiplex PCR, and identified 114 pathogens associated with BSIs in a few hours (Han et al., 2024). Moragues-Solanas et al. (2024) have demonstrated that nanopore sequencing of spiked blood samples with sepsis-relevant pathogens can identify bacteria and antibiotic resistance determinants in under 12 h. In our earlier study, we demonstrated that nanopore sequencing can detect sepsis-causing pathogens and their ARGs within 7 to 9 h (Ali et al., 2024).

Although nanopore sequencing shows promise for rapid BSI diagnosis, it requires the extraction of pure and high-molecular-weight bacterial DNA. However, DNA extraction from blood samples faces several challenges. The number of circulating microbes in BSIs is usually low (1–100 CFU/mL), and the host DNA significantly exceeds the microbial DNA due to leukocytosis (Forbes et al., 2017; Poole et al., 2018; Belok et al., 2021). The typical number of white blood cells (WBCs) in human blood is usually 4,500 to 11,000 cells/μL (El Brihi and Pathak, 2025). Additionally, the human genome comprises approximately 6 billion bases, whereas the average bacterial genome is roughly 3.5 million bases (Matsushita et al., 2014; Nurk et al., 2022). In a cell-to-cell comparison of genome content, the total nucleic acid in a bacterium (3.5 femtograms/cell) makes up only about 0.06% of a single human cell’s DNA (6 picograms/cell) (Islam Sajib et al., 2024). Due to these complexities, extracting sufficient bacterial DNA from blood samples for detection by nanopore sequencing remains a significant challenge.

To address the challenge of high human DNA levels and low microbial DNA in clinical samples, several studies have employed selective host cell lysis using non-ionic detergents, such as saponin, Triton X-100, and Tween-20 (Hasan et al., 2016; Charalampous et al., 2019; Shi et al., 2022; Moragues-Solanas et al., 2024; Bellankimath et al., 2025). Specifically, saponin, which targets cholesterol-rich plasma membranes, has been investigated for the selective lysis of host cells in clinical samples, such as blood, urine, and sputum (Zheng and Gallot, 2020; Alcolea-Medina et al., 2024; Charalampous et al., 2024; Moragues-Solanas et al., 2024; Bellankimath et al., 2025). This process of selective host cell lysis releases DNA, which is then removed in a subsequent step. Chemical methods, such as propidium monoazide, and enzymatic methods based on endonuclease enzymes (like DNase I/II, benzonase, and salt-activated nucleases), have been used to deplete host DNA before total DNA extraction for downstream applications (Marotz et al., 2018; Charalampous et al., 2019; Heravi et al., 2020). Recently, heat-labile salt-activated endonucleases (HL-SAN) and medium salt-activated endonucleases (M-SAN) have been studied for digesting the released host DNA in sputum and urinary tract infection (UTI) samples (Alcolea-Medina et al., 2024; Bellankimath et al., 2025). HL-SAN is optimized for digesting nucleic acids under high-salt conditions. The added salt also enhances chromatin disruption, which accelerates host depletion by this enzyme (Mayer et al., 2023; ArcticZymes Technologies, 2025). Meanwhile, M-SAN breaks down nucleic acids under physiological salinity conditions, enabling the detection of a broad range of bacterial and viral pathogens (ArcticZymes Technologies, 2025). The concentrations of saponin and enzymes, and the optimal conditions for enzyme activity, are essential for the selective and effective depletion of host DNA. Although the saponin concentration has been examined for targeted host cell lysis in blood, there has been limited research on optimizing SAN concentrations and salt conditions to achieve maximum endonuclease activity (Moragues-Solanas et al., 2024).

In this study, we aim to optimize host DNA depletion in blood cultures by testing different concentrations of saponin and SAN enzymes under various salt conditions (NaCl and MgCl_2_). Different concentrations of saponin were used to assess the effect of high saponin concentration on bacterial viability and cytolytic potential. Previous studies have evaluated HL-SAN exclusively for host DNA digestion in blood cultures (Moragues-Solanas et al., 2024). However, to our knowledge, this study is the first to assess M-SAN performance in removing host DNA from blood culture samples. Additionally, we tested different bead beating (BB) times for bacterial DNA extraction to recover longer DNA fragments suitable for nanopore sequencing. We found that 4% saponin is optimal for selectively lysing host cells, followed by digestion of the released DNA using 10 μL (250 U) of SAN enzymes. The different salt concentrations tested did not significantly affect enzyme activity. A shorter BB time of 6 min was more effective at recovering longer DNA fragments suitable for nanopore sequencing compared to a 10-min BB.

## Methods

### Bacterial strains

In this study, two of the most common bacterial pathogens that cause sepsis, *Escherichia coli* and *Staphylococcus aureus*, were used for testing. The *E. coli* isolate, identified as NCTC 13441, was obtained from the National Collection of Type Cultures (NCTC), Public Health England. Similarly, *S. aureus* CCUG 17621 was sourced from the Culture Collection of the University of Gothenburg (CCUG, Sweden). The *E. coli* NCTC 13441 is an extended-spectrum-beta-lactamase (ESBL) positive resistant isolate that has been tested in our previously published studies (Ali et al., 2024; Bellankimath et al., 2024). The *S. aureus* CCUG 17621 isolate is a wild type with no phenotypic resistance reported. These bacterial strains were stored at −80 °C in glycerol and refreshed on agar plates 1 day before the experiments. After testing and optimization with *E. coli* and *S. aureus* blood cultures, the final protocol was validated using other sepsis-related pathogens, including *Enterococcus faecium*, *Klebsiella pneumoniae* CCUG 225T, *Pseudomonas aeruginosa* CCUG 17619, and *Enterococcus faecalis* CCUG 9997.

### Testing of saponin concentration for selective lysis of host cells and preservation of bacterial cells

A range of saponin (Sigma-Aldrich, Catalog no. 47036-50G-F) concentrations was tested to achieve blood cell lysis without affecting bacterial viability. Bacterial growth dynamics were used to assess cell viability after saponin treatment. Therefore, bacterial growth was measured using the standard microdilution method (Theophel et al., 2014). Before measuring growth kinetic parameters, bacterial strains were prepared as follows.

**Figure 1:**
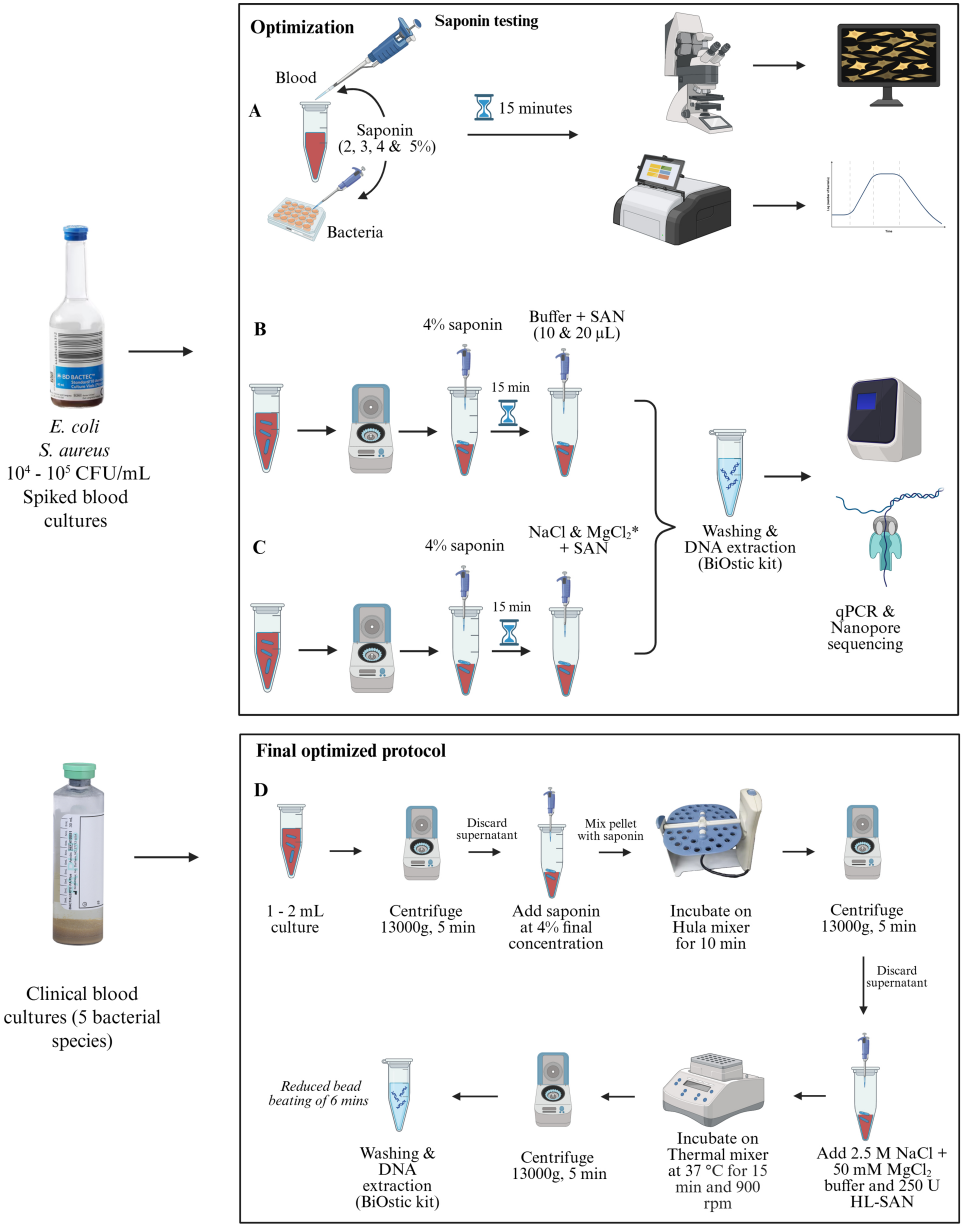
Overview of the experimental design. **(A)** Testing different concentrations of saponin (2%–5%) to observe the lysis of host cells and bacterial cells. **(B)** Host DNA depletion of *Escherichia coli* and *Staphylococcus aureus* blood cultures using saponin and various concentrations of salt-activated endonucleases (SAN), followed by DNA extraction. **(C)** Assessing the effect of different salt concentrations on SAN activity. **(D)** The final optimized protocol uses 4% saponin, 10 μL (250 U) of HL-SAN, 2.5 M NaCl, and 50 mM MgCl_2_ for effective host DNA depletion. * indicates methodology where the following concentrations were tested: 2 vs. 2.5 M NaCl + 50 mM MgCl_2_, 2.5 M NaCl + 15 vs. 50 mM MgCl_2_, 0.5 M NaCl + 15 mM MgCl_2_ vs. 2.5 M NaCl + 50 mM MgCl_2_ (Created with BioRender.com).

Bacterial strains were revived from glycerol stock and isolated as single colonies from an overnight static growth culture on Brain Heart Infusion (BHI) agar medium (VWR Life Sciences). An individual colony from each strain was inoculated into 3 mL of BHI broth and cultured for 2–3 h with moderate shaking at 37 °C, depending on the strain’s background. After brief cultivation, the culture samples were centrifuged at 2000 *g* for 10 min. The supernatant was discarded, and the pellets were resuspended in Phosphate-Buffered Saline (PBS) (VWR Life Sciences). Bacterial cell density was adjusted to 10^5^ CFU/mL and seeded into each well of a 96-well plate at the final concentration. Saponin was added to the wells at a final concentration of 2%–5%, with increments of 1%. The Breathe-Easy sealing membrane (Z380059, Merck) was used to seal the plates, which were then incubated at 37 °C in a Synergy H1 microplate reader. Absorbance at 600 nm for each well was measured at 30-min intervals over 24 h (Figure 1A).

### Growth data analysis using QurvE

Acquired growth data was analyzed using QurvE software v1. Raw data generated from the microplate reader was transformed to fit QurvE, and parameters such as growth OD threshold, time at 0 h (t0), and maximum growth time (tmax) were set during quality checks on the raw growth curve. In this study, the growth threshold was set to 1.5, and tmax to 24 h to extract growth kinetics parameters. Growth profiling of individual bacteria was performed using a growth-fitting exponential growth model with a heuristic linear regression on log-transformed data (Hall et al., 2014). Growth rates (*μ*) were measured for individual bacteria cultivated in growth medium with and without saponin (PBS used as a negative control). A linear fit was performed using an R_2_ threshold of 0.95, a relative standard deviation (RSD) of 0.1, and a dY threshold of 0.05 to estimate differences in maximum and minimum growth. The growth rate obtained from the linear fit model was validated with the Gompertz parametric fit model. Additionally, the doubling time of growth kinetics was measured on log-transformed data using a spline fit model with a smoothing factor set to 0.55 (Kahm et al., 2010). On the other hand, to validate the planktonic growth status in the presence of saponin, the static growth of bacteria was qualitatively validated using the droplet CFU method. After incubation in the presence of saponin at room temperature for 10–15 min, 10 μL droplets with three replicates from each saponin concentration were plated on BHI-agar plates and incubated overnight at 37 °C. Initial stock concentrations were approximately 10^5^ CFU/mL. Control samples with no saponin treatment were also plated out and compared to the treated samples for the effect of saponin on bacterial growth. Additionally, brightfield imaging was performed briefly to monitor the lysis of erythrocytes, providing supporting evidence of host cell depletion. Brightfield images of a blood sample treated with a targeted saponin concentration were obtained using a microscope (Nikon Eclipse Ti) at 200x magnification.

### Preparation of bacterial inoculum and blood culturing

The bacterial isolates were revived from glycerol stocks by streaking onto BHI agar medium. The bacterial inoculum was prepared as previously described, with minor modifications (Ali et al., 2024). Briefly, three to four colonies from overnight-grown bacterial cultures were suspended in fresh BHI broth and incubated for 2 hours. After incubation, the cultures were centrifuged, and the supernatant was discarded. The bacterial pellet was then resuspended in PBS, and the absorbance was measured at an optical density (OD) of 600 nm using a spectrophotometer (Thermo Electron Corporation, BioMate 3, USA). Dilutions of these suspensions were made to achieve 1,000 CFU/mL, and 1 mL was added to 10 mL of defibrinated sheep blood (ThermoFisher Scientific). Along with spiking the sheep blood, these suspensions were cultured on agar plates and incubated overnight to determine the colony-forming units per mL (CFU/mL). The spiked blood samples were then inoculated into BD BACTEC™ Plus Aerobic medium (BD) culture bottles and incubated at 37 °C. Bacterial growth in the blood culture was monitored by drawing blood samples hourly and plating them onto BHI agar. The aim was to obtain blood cultures with 10^4^ – 10^5^ CFU/mL. In addition to culturing the blood samples on BHI agar plates, aliquots were stored at −20 °C. Samples confirmed to have 10^4^–10^5^ CFU/mL were later used for DNA extraction.

### Optimization of SAN for host DNA depletion

#### Concentration of the SAN nucleases

The host DNA depletion in blood cultures was carried out by treating the blood cultures with 4% saponin, followed by the addition of HL-SAN (Catalog no. 70910-202) and M-SAN (Catalog no. 70950-202) (ArcticZymes Technologies, Norway). Before adding endonucleases, a specific buffer containing 2.5 M NaCl and 50 mM MgCl_2_, with pH levels of 7 and 8.3 for M-SAN and HL-SAN, respectively, was used. In this study, we used two different concentrations, 250 U (10 μL) and 500 U (20 μL), of both HL-SAN and M-SAN, to evaluate their performance for host DNA depletion in blood cultures. Our goal was to determine whether lower concentrations of SAN nucleases could achieve host depletion efficiency similar to that of higher concentrations (Figure 1B).

### Testing various salt concentrations for SAN activity

After optimizing endonuclease concentrations, various salt combinations were tested to prepare the enzyme buffer, evaluating their effects on enzyme activity and host DNA depletion.

*2 and 2.5 M NaCl with 50 mM MgCl_2_*: Initially, the buffer was prepared in nuclease-free water at a concentration of 2.5 M NaCl and 50 mM MgCl_2_ (pH 8.3 for HL-SAN and 7 for M-SAN), as previously reported (Bellankimath et al., 2025). The enzyme activity was evaluated for host depletion using this buffer and then compared with a lower concentration of 2 M NaCl, while maintaining a consistent concentration of MgCl_2_ (50 mM).

*15 and 50 mM MgCl_2_ with 2.5 M NaCl*: The NaCl concentration in the buffer was maintained at 2.5 M, and two different concentrations of MgCl_2_ (15 mM and 50 mM) were tested.

*0.5 M NaCl and 15 mM MgCl_2_ vs 2.5 M NaCl and 50 mM MgCl_2_*: Finally, a different buffer combination, using 0.5 M NaCl and 15 mM MgCl_2_, was also examined and compared with the other previously tested salt concentrations. All these salt combinations were evaluated for their ability to deplete host DNA by SAN in both *E. coli* and *S. aureus* blood cultures (Figure 1C). The effectiveness of the enzymes for host DNA depletion at various salt concentrations was assessed using either qPCR, sequencing, or both, as described in the following sections. For each salt-condition assay, two biological replicates were analyzed.

### DNA extraction and quality assessment

After selectively depleting host DNA with saponin and SAN endonucleases, all samples underwent DNA extraction. The QIAamp BiOstic Bacteremia Kit (Catalog no. 12240-50, Qiagen, Germany) was used for extraction, following the previous protocol with minor modifications (Taxt et al., 2020; Ali et al., 2024). Briefly, the pellet obtained after selective host depletion was washed twice with PBS to remove residual contaminants, then treated with lysis buffer from the BiOstic kit, followed by DNA extraction. In addition to the DNA extractions from host-depleted samples, a subset of samples was also extracted using the standard BiOstic kit protocol, which excluded the host depletion step involving saponin and SAN.

The quantification of all extracted DNA samples was performed using the Qubit 4 fluorometer with the Qubit 1X dsDNA HS Assay Kit (Catalog no. Q33231, ThermoFisher Scientific). The purity of the extracted DNA was assessed with a Nanodrop ND-1000 spectrophotometer (ThermoFisher Scientific), which measured absorption ratios at wavelengths of 260/280 nm and 260/230 nm. DNA fragment size distribution and integrity were evaluated using the Agilent 4,150 TapeStation System with the Genomic DNA ScreenTape assay (Catalog no. 5067-5,365, 5,067-5,366, Agilent Technologies, USA).

### Optimization of the bead beating protocol

The BiOstic kit employs a combination of chemical and mechanical methods to lyse cells and release DNA. The manufacturer’s default protocol recommends 10 minutes of continuous beating after adding lysis buffer to the sample. In these experiments, we employed three cycles of 2-min bead beating sessions, with a 1-min incubation on ice between each cycle. The bead beating was performed using a VWR Vortex laboratory shaker (444-2790) at 2500 rpm (15,708 rad/min). The samples processed with the reduced bead beating protocol were compared to those from the default protocol to evaluate any differences in DNA fragment lengths (as measured by TapeStation) and their impact on sequencing results.

### Method testing using clinical blood culture and additional sepsis relevant pathogens

The final optimized protocol was further tested on five clinical blood culture samples from sepsis patients. The positive blood culture samples were provided by University Hospital of North Norway, Tromsø. The BacT/ALERT (bioMérieux) was used for incubation, and the bottles were taken out once flagged positive by the system. The bottles were processed as a routine clinical diagnostic and aliquot of the blood cultures were provided for DNA extraction. These five samples were identified by clinical routine data as *E. coli*, *S. aureus*, *K. pneumonie*, *E. faecium*, and *P. aeruginosa*. Along with clinical samples four spiked samples using additional sepsis-causing pathogens such as *K. pneumonie*, *E. faecalis*, *E. faecium*, and *P. aeruginosa* were also validated using the final optimized protocol. The protocol involves using 4% saponin for selective lysis of host cells, followed by depletion with 250 U of HL-SAN combined with a buffer containing 2.5 M NaCl and 50 mM MgCl_2_ (Figure 1D).

### qPCR analysis

All extracted DNA samples were evaluated for the relative proportions of host (*Ovis aries*) and bacterial DNA. For quantifying bacterial and host DNA, species-specific primers were used (Supplementary Table 1). The qPCR reactions were performed using a 7,500 Fast Real-Time PCR system (Invitrogen™, USA) and QuantStudio™ 5 (ThermoFisher Scientific). Each PCR reaction included 3 *μ*L of 5X HOT FIREPol® EvaGreen® qPCR Supermix (Catalog no. 08-36-00001, Solid BioDyne, Estonia), 0.2 μM of forward and reverse primers, 10.4 μL of nuclease-free water, and 1 μL of template DNA. The qPCR amplification conditions were as follows: initial denaturation at 95 °C for 12 min, 40 cycles of 95 °C for 25 s, 60 °C for 45 s, and 72 °C for 60 s, with a final dissociation stage. Host DNA depletion and bacterial DNA enrichment were assessed using ΔCt values, normalized to the Ct of the reference control samples. These normalized Ct values were converted to fold changes using the equation 2^−ΔCt^. The fold changes were represented on a log scale to improve visualization. qPCR was performed with three technical replicates for each biological replicate. For salt assays, statistical analysis was conducted using a two-tailed unpaired t-test to assess differences between experimental groups.

### Nanopore sequencing and bioinformatics analysis

Some of the extracted DNA samples were sequenced using nanopore sequencing (Oxford Nanopore Technologies, UK). The sequencing was performed with the MinION MK1D device and an R10.4.1 flow cell (FLO-MIN114). Library preparation for sequencing was conducted using the Rapid Barcoding Kit 96 V14 (SQK-RBK114.96) and the Rapid PCR Barcoding Kit 24 V14 (SQK-RPB114.24).

Raw sequencing data were obtained using the ONT MinKNOW GUI software (Version 6.0.11), and fast basecalling was performed in real time with the Dorado basecaller. Unclassified reads were recovered with an in-house tool, MysteryMaster (Khezri et al., 2025). The identification of bacterial pathogens from the sequencing data was carried out as previously described (Bellankimath et al., 2025). Briefly, the basecalled sequencing data were BLAST searched against a custom reference database of the most common sepsis-causing pathogens. Reads that did not align with any prokaryotic genome were presumed to be host reads. To evaluate the percentage of mitochondrial DNA in the host DNA, raw reads were also BLAST searched against the *Ovis aries* mitochondrial reference genome (NC_001941) from the NCBI database.

## Results

### 4% saponin selectively lysed host cells while leaving bacterial cells intact

**Figure 2:**
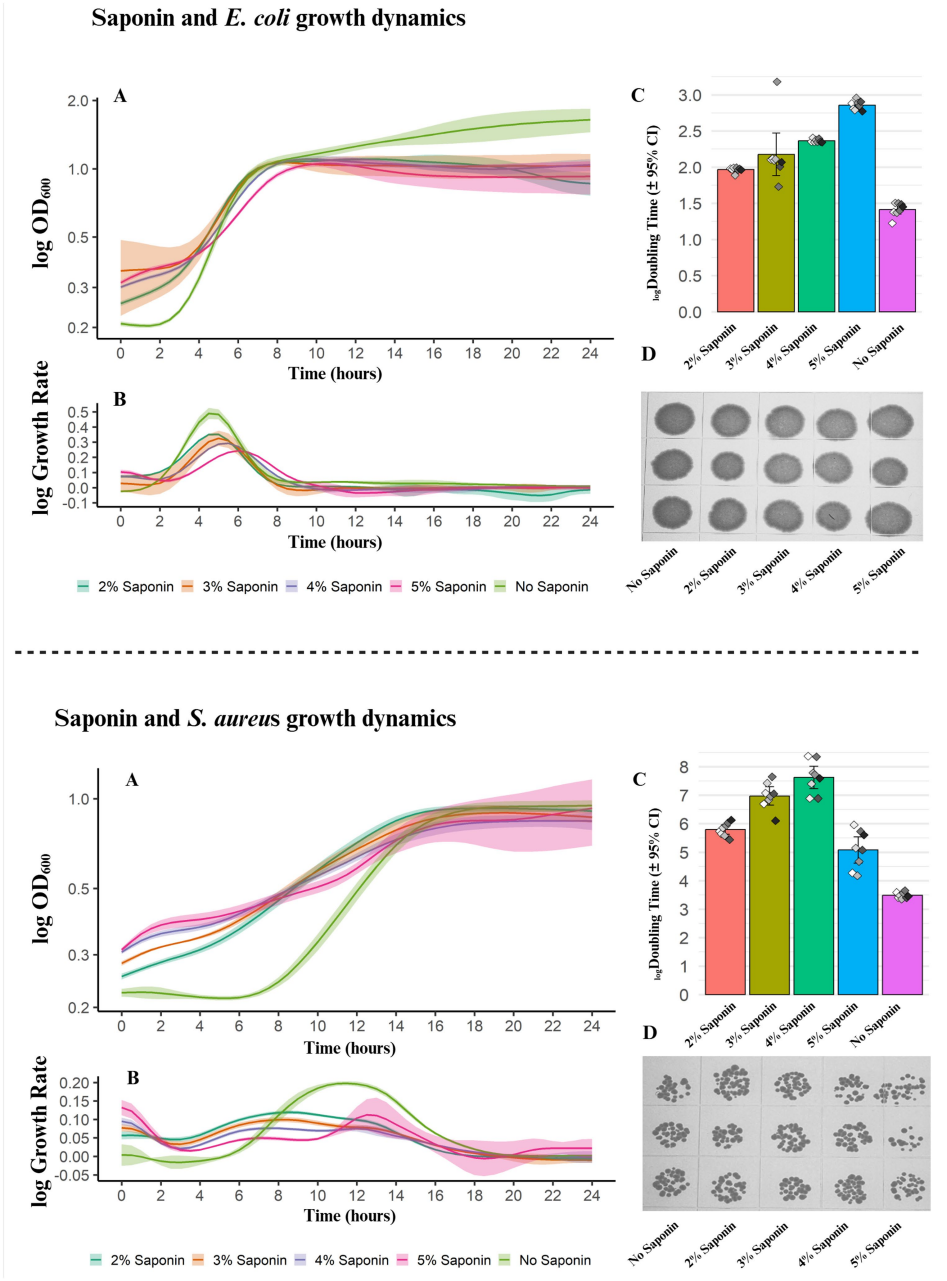
Effect of saponin on bacterial growth dynamics (*Escherichia coli* and *Staphylococcus aureus*). For both strains **(A)** Growth curves over time (24 h) for conditions with control (PBS), 2–5% saponin. Growth curves depicting log-transformed OD600 on the y-axis and hours on the x-axis. **(B)** Smoothed line histogram demonstrating the distribution of growth rates across saponin conditions, with overlapping curves indicating variability. **(C)** Doubling time (log transformed), highlighting differences in growth kinetics. Bar plot with confidence interval (CI-95%) for each condition depicting gradual changes in doubling across the saponin concentration. **(D)** Static growth images on BHI agar for both strains illustrate no effect of saponin on colony formation on agar.

The results showed that control (without saponin) had the highest planktonic growth rate, measured by growth rate (μ) using a linear fit model and 95% confidence interval (CI). The growth rate gradually declined as saponin concentration increased, indicating a dose-dependent inhibition (Figure 2). At 2%–4% saponin, *E. coli* and *S. aureus* displayed similar growth patterns, suggesting minimal differences between these concentrations. However, a marked decrease in planktonic growth was observed at 5% saponin, signaling the start of an inhibitory effect at this higher concentration. Growth trends observed in the curve also reveal that the growth rate increases over time, peaking between 4 and 6 h for *E. coli*. The control sample exhibits the highest peaks, followed by the saponin-treated samples, which reinforces the impact of saponin on bacterial growth. *S. aureus* showed a rapid decline in growth rate at the start of saponin treatment but gradually recovered after 4 h, demonstrating cellular adaptability and viability under dysbiosis conditions (Figures 2A,B). Nonetheless, the control samples reached the highest peak after 6 h and then stabilized after 16 h for *S. aureus*.

Doubling times measured for both *E. coli* and *S. aureus* support the growth rate patterns exerted by saponin (Figure 2C). *E. coli* samples without saponin treatment had the shortest doubling time, whereas those treated with 5% saponin exhibited the longest doubling time. Interestingly, in *S. aureus*, although no saponin had the shortest doubling time, samples with 5% saponin had the second shortest, likely due to viability dysbiosis. *S. aureus* typically exhibits a slower doubling time. During the first 4–6 h without saponin, the lag phase reflects the typical growth dynamics of *S. aureus* in BHI media. However, the addition of saponin, which slightly darkens the contrast of the BHI media, elevates the optical density (OD) at time zero. In the presence of saponin, *S. aureus* undergoes some metabolic changes, as indicated by the fluctuations in OD over time for each concentration of saponin. After 14–15 h, the saponin-treated samples enter the stationary phase. This suggests that individual concentrations of saponin affect the growth dynamics of *S. aureus*. To validate this effect, we conducted calculations of the doubling time and performed agar static growth experiments to elucidate the true biological impact of saponin on the bacteria.

Additionally, static growth in the presence of saponin suggests non-significant inhibition of cell viability. Qualitative droplet-based static growth assays show steady bacterial colony growth on BHI (brain heart infusion) agar, except for *S. aureus* at 5% saponin, where fewer colonies were observed (Figure 2D). This suggests that saponin levels above 5% may inhibit bacterial growth. Microscopic examination of saponin-treated blood samples also indicates that saponin selectively affects host cells rather than bacteria at the tested concentrations (Supplementary Figure 1). In this study, 4% of saponin was used, and blood samples treated with saponin show erythrocyte lysis. Overall, *E. coli* and *S. aureus* appear to tolerate saponin concentrations of 2%–5% well, allowing for the complete lysis of blood cells, except for platelets. To maintain the downstream experimental workflow, 4% saponin was chosen as it selectively lyses host blood cells and supports both static and planktonic bacterial growth.

### Blood cultures reached 10^4^–10^5^ CFU/mL after 2 to 4 h of incubation

To optimize the bacterial inoculum for blood culturing, absorbance at OD 600 nm was measured. Overnight incubated *E. coli* and *S. aureus*, grown for 2 hours in fresh BHI with an absorbance of 0.1 and 0.2 at OD 600, reached a concentration of 10^7^ CFU/mL. This suspension was further diluted to achieve a final concentration of ~100 CFU/mL in blood. The *E. coli* spiked blood cultures showed ~10^5^ CFU/mL after 2 hours of incubation and reached 10^6^ CFU/mL after 4 hours of incubation. Similarly, the *S. aureus* blood culture showed ~10^2^ CFU/mL at 2 hours of incubation and reached 10^4^ CFU/mL after 4 hours.

### 250 Units of SAN demonstrated similar host depletion as 500 units

**Figure 3:**
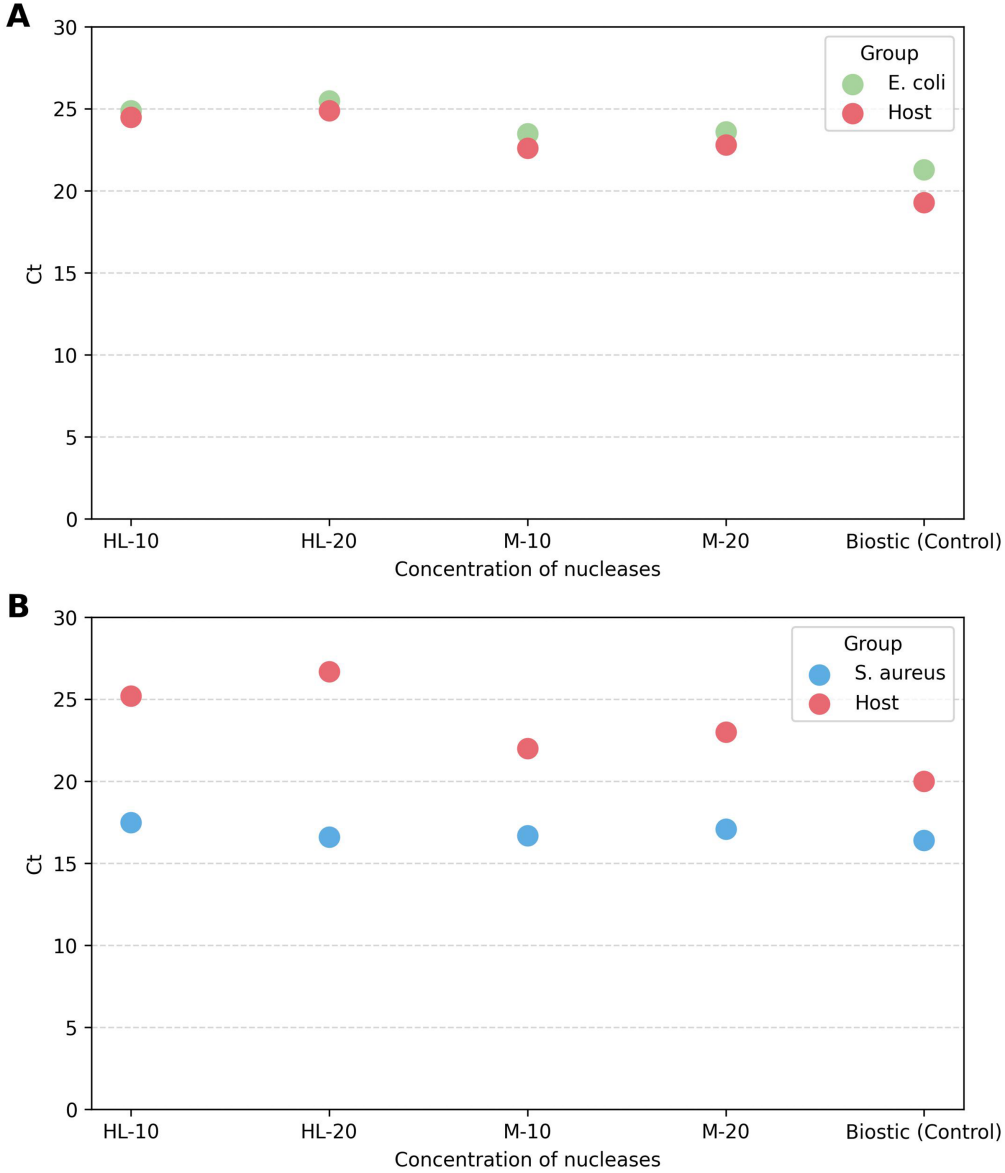
Effect of different enzyme concentrations on host DNA depletion evaluated using qPCR Ct values. **(A)** *Escherichia coli* **(B)** *Staphylococcus aureus*. HL-10 / HL-20 = 10 or 20 μL (250/500 U) HL-SAN, M-10 / M-20 = 10 or 20 μL (250/500 U) M-SAN.

The qPCR analysis of bacterial and host DNA, extracted after host depletion with two different concentrations of HL-SAN and M-SAN, revealed that 250 U (10 μL) of SAN produced results comparable to those obtained with 500 U (20 μL) in terms of host DNA depletion and bacterial DNA recovery (Figure 3).

For *E. coli* blood cultures, HL-SAN at both concentrations (250 and 500 U) produced very similar Ct values (24.5 vs. 24.9) for host primers, indicating comparable host DNA depletion (Figure 3A). In contrast, the average bacterial Ct values were 24.9 at 250 U and 25.5 at 500 U, suggesting better bacterial DNA recovery at the lower HL-SAN concentration (250 U). M-SAN for host primers showed an average Ct of 22.6 at 250 U and 22.8 at 500 U, suggesting similar host DNA depletion. Meanwhile, for the bacterial primers, the average Ct values were 23.5 at 250 U and 23.6 at 500 U. *Escherichia coli* samples extracted using the standard BiOstic protocol without depletion displayed lower average Ct values of 21.3 for bacteria and 19.3 for host (Figure 3A).

In *S. aureus* blood cultures, HL-SAN and M-SAN showed similar average Ct values of ~17 for bacterial primers at all tested concentrations. However, host primer Ct values were 26.7 and 25.2 with HL-SAN at 500 and 250 U, respectively, indicating slightly better host DNA depletion at the higher nuclease concentration (Figure 3B). M-SAN produced comparable results, with higher enzyme levels resulting in increased host Ct values (average Ct of 23) compared to lower levels (average Ct of 22). The *S. aureus* non-depleted (BiOstic protocol) samples showed an average Ct of 20 for the host primers and 16.4 for bacterial primers.

Overall, both enzymes demonstrated lower Ct values for bacterial primers than for host primers, reflecting effective host depletion. The slight difference in host Ct values between *E. coli* (24.9) and *S. aureus* (26.7) is because these are two separate blood cultures prepared from a single batch of sheep blood. The ~1.8 cycle difference is due to sampling and stochastic variation, or to variation arising during handling. Host depletion was evaluated against the reference BiOstic kit protocol, which did not include depletion. The host Ct values for samples extracted only with the BiOstic kit, without depletion, were lower (average Ct = 20), indicating a greater amount of host DNA.

In *E. coli* blood cultures, using a lower concentration of HL-SAN and M-SAN (250 U) for host depletion produced a higher DNA yield (55 and 105 ng) compared to the higher enzyme concentration (500 U) (45 and 90 ng), respectively. Similarly, *S. aureus* samples showed high DNA yield (230 ng) when M-SAN (250 U) was used for depletion. However, in HL-SAN samples, the higher concentration (500 U) resulted in a greater DNA yield (115 ng) (Supplementary Table 2).

### SAN nuclease activity stayed stable across different concentrations of NaCl and MgCl_2_

*2 and 2.5 M NaCl with 50 mM MgCl_2_*: *E. coli* blood culture samples treated with both SAN enzymes (250 U) showed comparatively lower levels of host DNA depletion when 2 M NaCl was used in the buffer as opposed to 2.5 M NaCl. Samples processed with HL-SAN showed average Ct values of 21.9 and 24.5 for host primers at 2 M NaCl and 2.5 M NaCl, respectively. *E. coli* M-SAN showed a similar trend, yielding an average Ct value of 20.7 at 2 M NaCl and 22.6 at 2.5 M NaCl (Supplementary Figure 2A). Bacterial DNA recovery in *E. coli* samples was higher when HL-SAN was used with 2.5 M NaCl (average Ct = 24.9) than with 2 M NaCl (average Ct = 26.9). M-SAN-processed samples showed similar bacterial DNA recovery (average Ct = 23.5) under both tested conditions. *S. aureus* samples treated with HL-SAN showed comparatively similar host DNA depletion at both 2 M NaCl (average Ct = 25.4) and 2.5 M NaCl (average Ct = 25.2). However, *S. aureus*, while using M-SAN, showed a higher level of host DNA depletion at 2 M NaCl (average Ct = 23.6) compared to 2.5 M NaCl (average Ct = 20) (Supplementary Figure 2B). Similarly, in *S. aureus* samples, bacterial DNA recovery was higher at 2 M NaCl (average Ct = 15.95) when using both HL-SAN and M-SAN compared to HL-SAN at 2.5 M NaCl (average Ct = 17.5) and M-SAN at 2.5 M NaCl (average Ct = 16.7). In both *E. coli* and *S. aureus*, higher DNA yields were obtained with HL-SAN and M-SAN at 2 M NaCl compared with 2.5 M NaCl (Table 1).

**Table 1:** Host depletion and DNA extraction from blood cultures using 2 and 2.5 M NaCl and 50 mM MgCl_2_ (constant) as buffers for enzyme activity.

Samples	Endonuclease used	Biological replicates	NaCl Conc (M)	DNA yield (ng)	Nanodrop quality check		Average CT (Bacteria)	Average CT (Host)
					260/280	260/230		
*E. coli*	HL-SAN	1	2	378	1.52	0.94	25.5	21.1
		2	2	218	1.24	0.83	27.7	22.2
		3	2	235	1.18	0.78	27.6	22.4
		Control	2.5	55	1.4	0.3	24.9	24.5
	M-SAN	1	2	520	1.44	0.76	22.9	20.1
		2	2	300	1.36	0.86	23.6	20.7
		3	2	190	1.29	0.63	23.9	21.4
		Control	2.5	105	1.5	0.2	23.5	22.6
*S. aureus*	HL-SAN	1	2	210	1.44	1.04	15.9	26.3
		2	2	235	1.54	1	16	24.5
		Control	2.5	75	1.3	0.1	17.5	25.2
	M-SAN	1	2	216	1.42	0.8	16.1	24.2
		2	2	291	1.51	0.81	15.8	23
		Control	2.5	230	1.6	1.1	16.4	20

**Figure 4:**
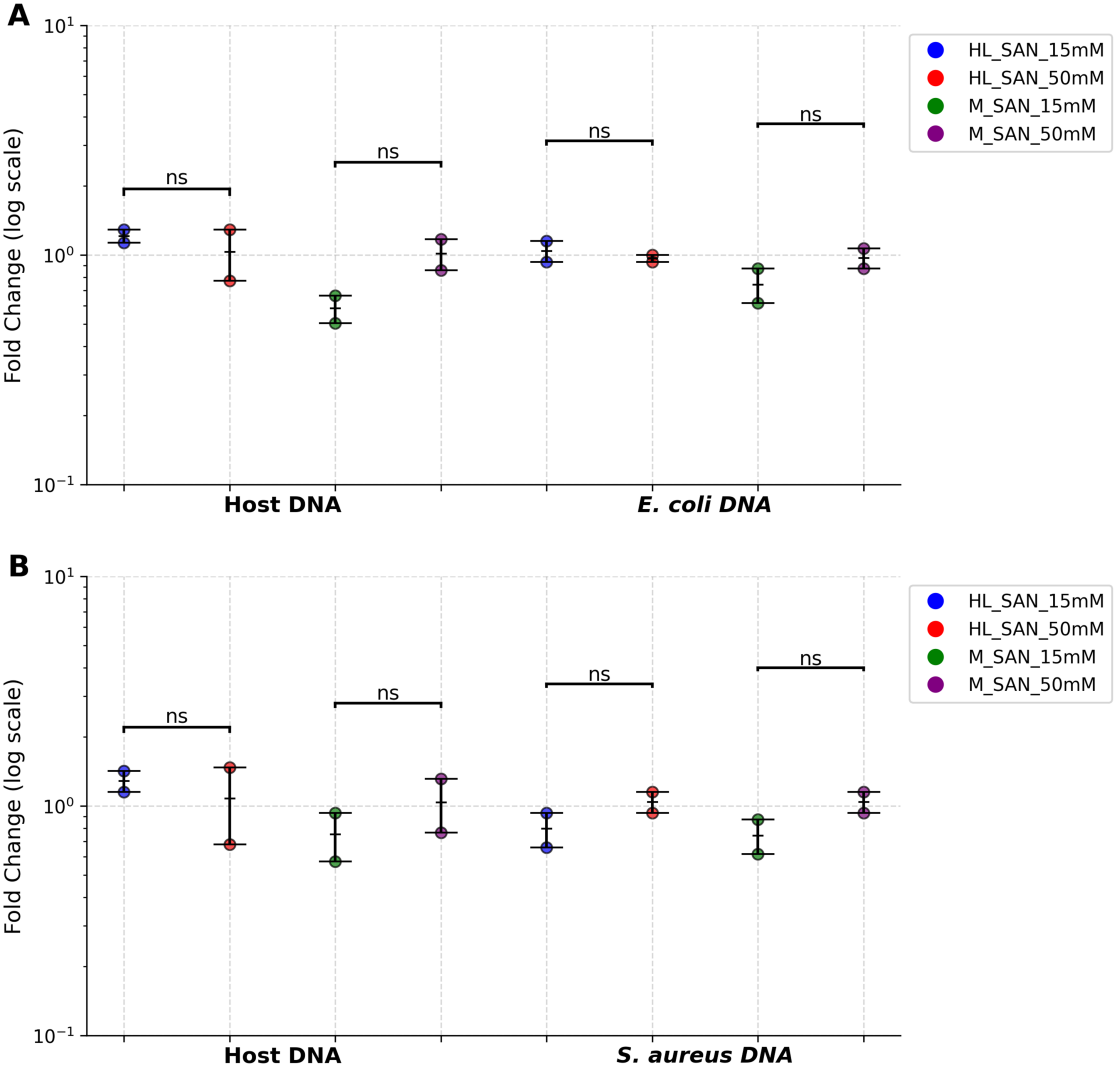
The effect of MgCl_2_ concentrations on HL-SAN and M-SAN activity for host DNA depletion. Host DNA depletion and bacterial DNA enrichment were assessed using ΔCt values, normalized to the control samples. These normalized Ct values were converted to fold changes and were represented on a log scale to improve visualization. The 50 mM MgCl_2_ concentration is used as a reference for comparison with the 15 mM MgCl_2_. The concentration of NaCl is kept constant at 2.5 M **(A)** *Escherichia coli* and **(B)** *Staphylococcus aureus*. 15 mM/50 mM = MgCl_2_. All test values are presented as mean ± SD, with n = 2 biological replicates for each condition. ns > 0.05; *p ≤ 0.05; **p ≤ 0.01; ***p ≤ 0.001; ****p ≤ 0.0001.

*15 and 50 mM MgCl_2_ with 2.5 M NaCl*: Different concentrations of MgCl_2_ (15 and 50 mM) were tested in the buffer, with a fixed NaCl concentration of 2.5 M (Supplementary Table 3). HL-SAN achieved similar host depletion (average Ct = ~23) and bacterial DNA (average Ct = ~19.5) recovery at both MgCl_2_ concentrations in *E. coli* blood cultures. *E. coli* samples processed with M-SAN showed slightly higher host depletion (average ΔCt = 0.8) at 15 mM MgCl_2_ compared to 50 mM, but bacterial DNA recovery also decreased (average ΔCt = 0.45) at 15 mM (Figure 4A). In *S. aureus* blood cultures, HL-SAN resulted in slightly less host DNA depletion and bacterial recovery (average ΔCt = 0.35) at 15 mM MgCl_2_ (Figure 4B). M-SAN at 15 mM MgCl_2_ resulted in higher host DNA depletion (average ΔCt = 0.45) than at 50 mM (Figure 4B). Overall, there were no significant differences in host DNA depletion or bacterial DNA recovery between the two enzymes at different MgCl_2_ concentrations in both *E. coli* and *S. aureus* blood cultures. In *E. coli*, higher DNA yields were obtained with HL-SAN at 15 mM MgCl₂ compared with 50 mM MgCl₂, whereas for M-SAN, higher DNA yields were observed at 50 mM MgCl₂. In *S. aureus* both HL-SAN and M-SAN yielded higher DNA at 50 mM MgCl₂ compared to 15 mM MgCl₂ (Supplementary Table 3).

**Figure 5:**
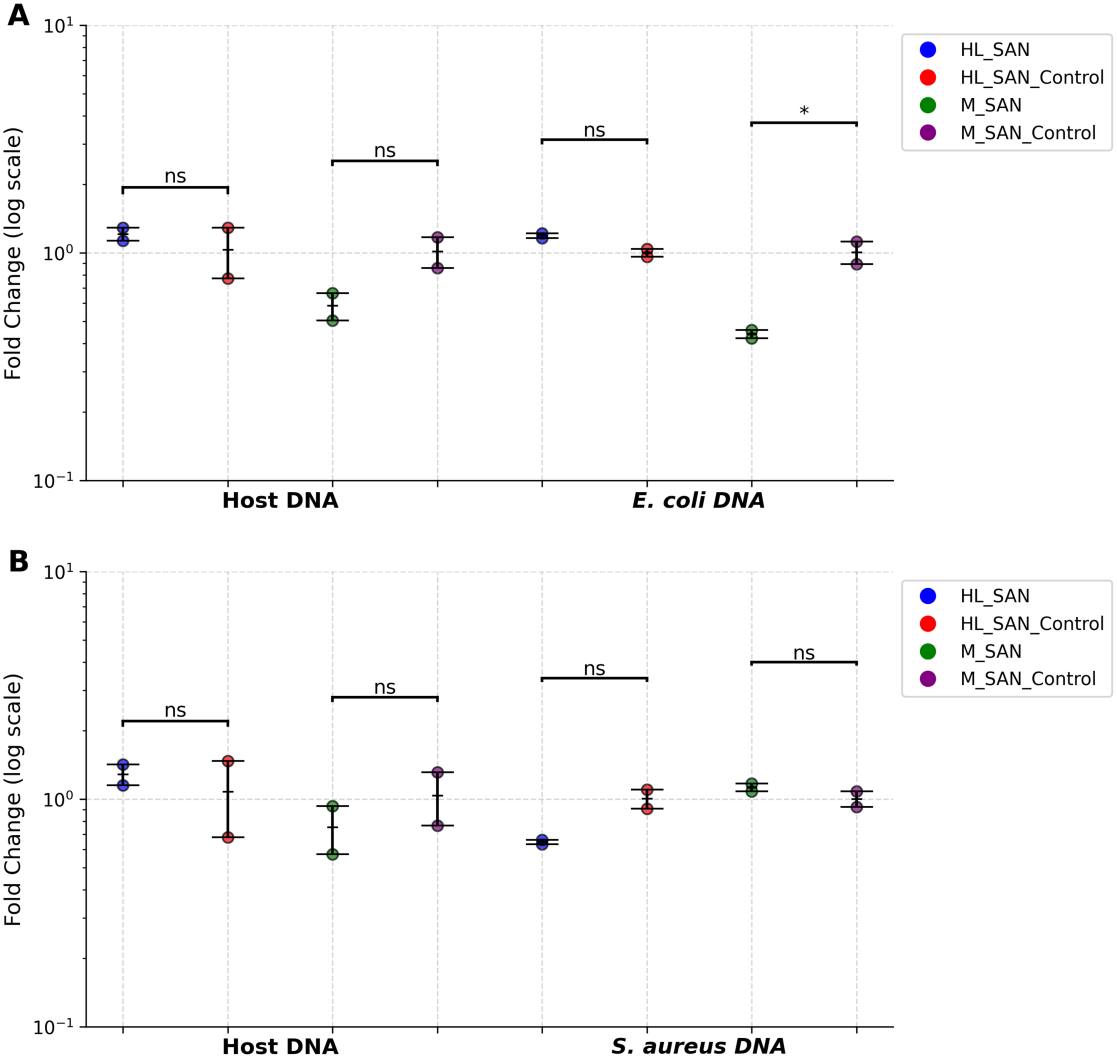
The effect of NaCl and MgCl_2_ concentrations on HL-SAN and M-SAN activity for optimal host DNA depletion. Host DNA depletion and bacterial DNA enrichment were assessed using ΔCt values, normalized to the control samples. These normalized Ct values were converted to fold changes and were represented on a log scale to improve visualization. The 2.5 M NaCl and 50 mM MgCl_2_ concentrations are used as reference control and are compared with 0.5 M NaCl 15 mM MgCl_2_. **(A)** *E. coli* **(B)** *S. aureus*. HL_SAN/M_SAN = 0.5 M NaCl and 15 mM MgCl_2_, HL_SAN/M_SAN control = 2.5 M NaCl and 50 mM MgCl_2_. All test values are presented as mean ± SD, with n = 2 biological replicates for each condition. ns > 0.05; *p ≤ 0.05; **p ≤ 0.01; ***p ≤ 0.001; ****p ≤ 0.0001.

*0.5 M NaCl and 15 mM MgCl_2_ vs 2.5 M NaCl and 50 mM MgCl_2_*: Reducing the NaCl concentration to 0.5 M and keeping MgCl_2_ at 15 mM, both enzymes (250 U) were evaluated for host depletion. The results from these conditions were compared with those from a control using 2.5 M NaCl and 50 mM MgCl_2_ (Supplementary Table 4). In HL-SAN *E. coli*, host DNA depletion was slightly less (average ΔCt = −1.25), with bacterial DNA recovery remaining similar at lower salt levels compared to the control. *E. coli* samples with M-SAN showed marginally higher host depletion (average ΔCt = 0.4) at lower salt levels, though a slight reduction in *E. coli* DNA (average ΔCt = 1.2) was also noted (Figure 5A). For *S. aureus* blood cultures treated with HL-SAN, host depletion was comparable, but bacterial DNA recovery was somewhat lower (average ΔCt = 0.7) at 0.5 M NaCl and 15 mM MgCl_2_. Similarly, *S. aureus* with M-SAN demonstrated slightly lower host DNA depletion (average ΔCt = −0.53) at lower salt conditions but the bacterial DNA recovery was similar at both salt concentrations (Figure 5B). In *E. coli* higher DNA yield was observed with HL-SAN at 0.5 M NaCl + 15 mM MgCl_2_ while M-SAN showed little difference in DNA yield between the two salt conditions. For *S. aureus,* HL-SAN yielded more DNA at 2.5 M NaCl + 50 mM MgCl₂, while M-SAN performed better at 0.5 M NaCl + 15 mM MgCl₂.

### Bead beating for 6 min enhanced the recovery of longer DNA fragments

To assess how reducing bead beating (BB) time to 6 min affects DNA fragment size and nanopore sequencing, blood cultures of *E. coli* and *S. aureus* were processed using the same DNA extraction protocol, except for the duration of BB. In the modified protocol, the usual 10-min continuous BB was replaced with three cycles of 2-min BB, each separated by a 1-min ice incubation. The extracted DNA samples were analyzed on the TapeStation and compared. Results showed that shorter BB enhanced the recovery of longer DNA fragments.

**Figure 6:**
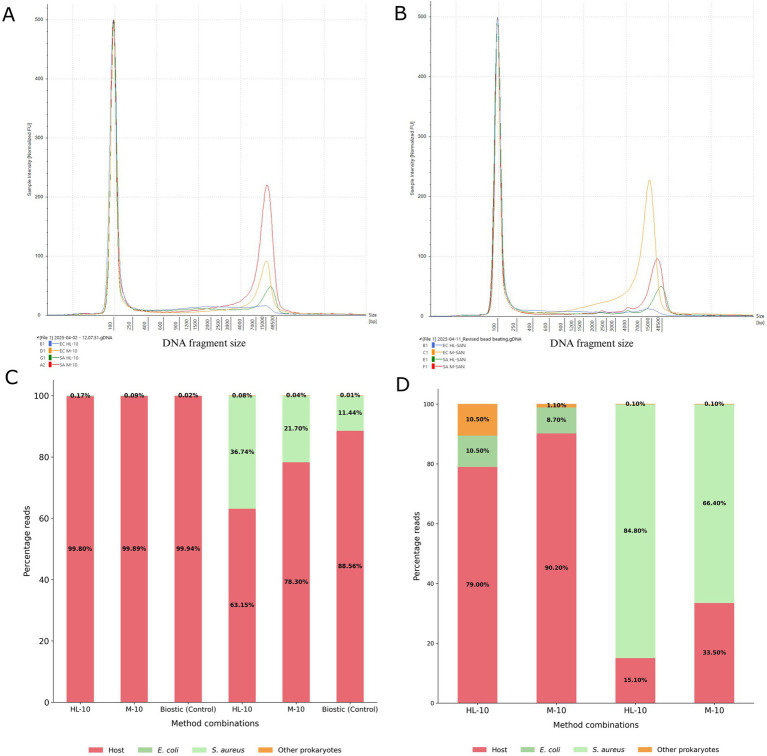
Fragmentation and sequencing results at 10 min and 6 min BB. The DNA fragment lengths were analyzed using TapeStation, and the sequencing reads generated by nanopore sequencing were analyzed for relative distribution of host and pathogen DNA. **(A)** 10 min BB fragmentation **(B)** 6 min BB fragmentation **(C)** 10 min BB sequencing reads distribution **(D)** 6 min BB sequencing reads distribution. EC = *Escherichia coli*, SA = *Staphylococcus aureus*, BB = bead beating.

DNA fragmentation analysis on TapeStation revealed that the *E. coli* sample processed with HL-SAN after 10 min of BB was highly fragmented, with the longest fragment of 2,110 bp at a concentration of 0.104 ng/μL (Figure 6A). When the BB time was reduced, a longer DNA fragment of 11,604 bp (0.234 ng/μL) was obtained (Figure 6B). Using M-SAN, the *E. coli* samples yielded a larger DNA fragment of 15,209 bp at 10 min of BB compared to a fragment of 13,210 bp obtained at reduced BB (6 min). However, the DNA concentration of the fragments was still higher at 6 min BB (10.2 ng/μL) compared to 10 min BB (3.23 ng/μL) (Figure 6).

*S. aureus* samples treated with HL-SAN showed two distinct DNA fragments of 1717 and 21,401 bp after 10 min of BB (Figure 6A). In contrast, samples processed with 6-min BB produced three longer DNA fragments of 2,251, 4,014, and 41,981 bp (Figure 6B). Similarly, *S. aureus* processed with M-SAN also displayed longer DNA fragments of 2,278, 4,010, and 23,739 bp with 6-min BB compared to the DNA fragment of 16,793 bp obtained after 10-min BB (Figure 6).

The sequencing results also indicated that using the default 10-min BB yielded fewer target pathogen reads compared to a shorter 6-min BB. In *E. coli* blood cultures with HL-SAN, M-SAN, and the BiOstic kit alone, followed by 10 min of BB, most of the extracted DNA was mapped to the host genome. *S. aureus* samples showed a higher percentage of bacterial reads, with 36, 22, and 11% of reads mapping to the target pathogen for HL-SAN, M-SAN, and BiOstic alone, respectively; the rest aligned with the host genome (Figure 6C). When BB time was shortened to 6 min, bacterial read numbers from nanopore sequencing increased notably*. E. coli* blood cultures produced 10.5% (HL-SAN) and 8.7% (M-SAN) of reads mapping to the target pathogen with the shorter BB protocol. Similarly, *S. aureus* samples showed an increased proportion of pathogen-specific reads, with 85 and 66% of total reads mapping to the target in HL-SAN and M-SAN treated samples, respectively (Figure 6D).

Moreover, higher average read lengths were observed in samples sequenced with 6-min BB compared to 10-min BB. In *E. coli*, the 10-min BB protocol yielded average read lengths of 466 bp and 1,024 bp for samples processed with HL-SAN and M-SAN, respectively. However, when the BB was reduced to 6 min, HL-SAN and M-SAN showed an increased average read length of 2,368 bp and 2,852 bp, respectively (Supplementary Table 5). Similarly, in *S. aureus*, 6 min of BB yielded longer average read lengths, with 3,542 bp for the HL-SAN and 3,884 bp for the M-SAN processed samples. In contrast, the default BB protocol of 10 min resulted in shorter read lengths of 1,406 bp (HL-SAN) and 1,611 bp (M-SAN) (Supplementary Table 5).

### The optimized protocol performed efficient host depletion on clinical blood cultures

**Figure 7:**
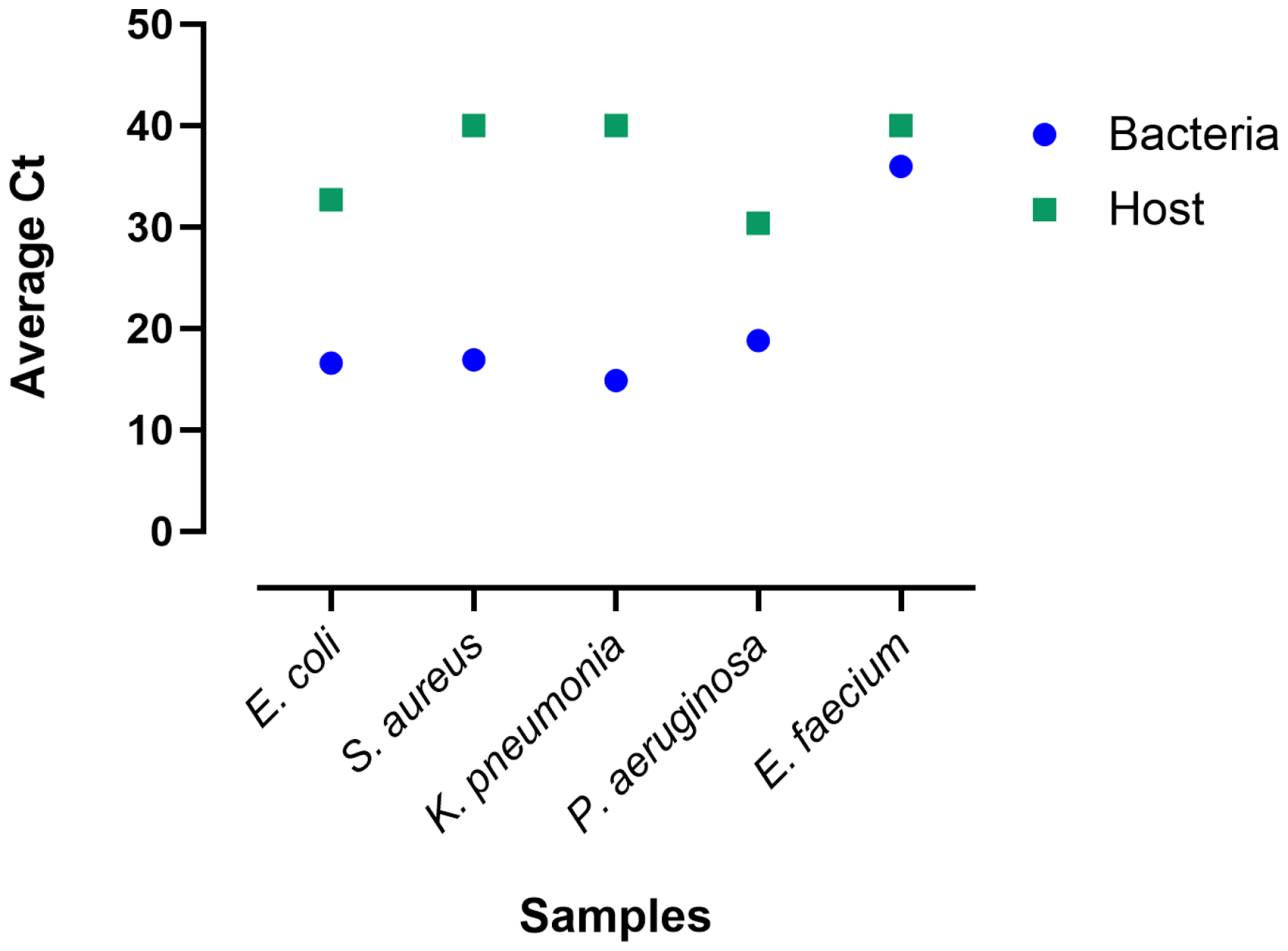
Validation of the final optimized protocol using clinical blood cultures from sepsis patients. qPCR average Ct values are presented using bacterial species-specific primers and host (human) primers.

For validation, the final optimized method was tested on five blood cultures flagged positive by the automated blood culture system and identified by MALDI-TOF during routine clinical testing. DNA was extracted from these blood cultures, and qPCR was performed. The results showed that the optimized protocol validated on *E. coli*, *K. pneumoniae*, and *E. faecium* provided a DNA yield of 11,550, 17,040, and 13,440 ng, respectively. In the other two blood cultures, *S. aureus* yielded 4,740 ng of DNA while *P. aeruginosa* provided the lowest DNA yield of 168 ng (Supplementary Table 6). The DNA obtained from these blood cultures will include both bacterial and human DNA. The qPCR results with species-specific primers for the DNA extracted from these clinical blood cultures showed efficient recovery of bacterial DNA and depletion of host DNA. *Escherichia coli* blood culture showed an average Ct of 16.6 for bacteria and 32.7 for host primers. *S. aureus*, *K. pneumoniae*, and *E. faecium* showed an average bacterial Ct of 16.9, 14.9, and 36, while the average host Ct for all these samples was approximately 40. *Pseudomonas aeruginosa* has an average Ct of 18.8 for bacteria and 30.4 for the host (Figure 7). The higher bacterial Ct for *E. faecium* might be due to the pipetting errors while normalizing the DNA for qPCR or loading the qPCR input.

The optimized protocol was tested on spiked blood cultures of the four sepsis-causing bacterial pathogens. The DNA extraction results showed that *E. faecium* yielded the highest amount of DNA (61.8 ng), followed by *K. pneumoniae* (55.5 ng). *P. aeruginosa* sample showed 32.4 ng DNA while *E. faecalis* showed the lowest amount of DNA yield, 12.6 ng (Supplementary Table 7). The qPCR results with the bacterial primers showed efficient recovery of bacterial DNA. The Ct values observed for *K. pneumoniae*, *E. faecalis*, *E. faecium*, and *P. aeruginosa* were 19.6, 24.6, 18.2, and 22.4, respectively (Supplementary Table 7).

## Discussion

Rapid diagnosis of BSIs and sepsis is essential to lower mortality rates and curb antibiotic resistance. Currently, sepsis diagnosis relies on conventional blood cultures combined with biochemical tests, MALDI-TOF MS, or qPCR detection (Costa and Carvalho, 2022). These methods can take up to 72 h to identify the causative pathogens and their associated antibiotic resistance (Briggs et al., 2021). Nanopore sequencing has the potential to overcome these challenges by providing a fast and accurate BSI diagnostic method (Taxt et al., 2020; Ahmadi et al., 2022; Ali et al., 2024; Bellankimath et al., 2024, 2025). One of the most challenging steps in sequencing-based diagnosis is extracting microbial DNA while minimizing host DNA contamination (Heravi et al., 2020). In this study, we developed a saponin-based selective lysis method for host cells and used HL-SAN and M-SAN enzymes to remove the released host DNA, followed by the extraction of bacterial DNA. Additionally, we tested various enzyme concentrations and salt levels to find the optimal conditions for depleting the host DNA. The results showed that 4% saponin, followed by 10 μL (250 U) of HL-SAN/M-SAN enzymes at various salt conditions, effectively depleted host DNA in *E. coli* and *S. aureus* blood cultures.

### 4% saponin is effective for the selective depletion of the host DNA

Chemically, saponins are amphipathic glycosides, featuring a hydrophobic aglycone (sapogenin) backbone, usually a triterpene or steroid, attached to one or more hydrophilic sugar groups. Due to this structure, saponins preferentially interact with cholesterol-rich membranes, a trait more common in eukaryotic cells than in bacterial cells (Moses et al., 2014). Both planktonic and static growth dynamics in the presence of saponin show that saponin is more likely to disrupt host cells by forming complexes with cholesterol, which causes pore formation in the host cell membrane, allowing osmotic imbalance and leakage of cellular contents. Bacterial cells, which lack cholesterol in their cell walls (mainly made of peptidoglycans), are less susceptible to this mechanism, which explains the observed selectivity. This compositional difference underpins the targeted action of saponin, making it an optimal agent for host cell lysis without significant effect on bacterial cells. Previous studies also support this selective lysis of saponin (Bruggeling et al., 2021; Narayana Iyengar et al., 2021). However, previous studies indicated that while saponin effectively depleted chromosomal DNA in blood samples, a significant amount of mitochondrial DNA remained (Moragues-Solanas et al., 2024). Since saponin acts on the cholesterol-rich membranes in eukaryotic cells, the outer and inner mitochondrial membranes contain relatively few cholesterol molecules (Horvath and Daum, 2013; Casares et al., 2019). Therefore, mitochondrial host cells are usually more resistant to lower concentrations of saponin treatment. Moragues-Solanas et al. (2024) observed that using 1% saponin for host depletion resulted in about 88% of the total host DNA coming from mitochondria. However, when the saponin concentration increased to 3%, the depletion of chromosomal and mitochondrial host DNA was enhanced 10-fold without affecting the bacterial DNA in the samples. These results align with ours, where we used 4% saponin and found that only around 0.1% of the total reads align with host mitochondrial DNA. The saponin and SAN-based method employed in this study effectively depleted the host chromosomal and mitochondrial DNA.

### Lower concentrations of endonucleases resulted in host depletion comparable to that at higher concentrations

In this study, two different concentrations of HL-SAN and M-SAN (250 U and 500 U) were used and compared to evaluate their efficiency in host DNA depletion in blood cultures. Using optimal enzyme concentration is crucial for effective host DNA depletion, as too little enzyme may be ineffective, while higher concentrations could negatively impact the downstream extraction process. Previous studies have also tested various enzyme concentrations for host DNA depletion in different sample types. Charalampous et al. (2019) initially used 5 + 2 μL of HL-SAN enzyme and later optimized host DNA depletion in respiratory samples by using 10 μL of HL-SAN enzyme (Djeghout et al., 2024). A similar 250 U (10 μL) of HL-SAN was used for the depletion of host DNA in stool, respiratory, and serum samples (Han and Xia, 2023; Alcolea-Medina et al., 2024; Djeghout et al., 2024). These studies have shown that host DNA depletion using 250 U (10 μL) of HL-SAN is effective. However, the sample types vary, and little research has focused on optimizing the enzyme concentrations for host DNA depletion in blood samples or cultures.

Moragues-Solanas *et al*. have demonstrated that using 250 U (10 μL) of HL-SAN with saponin-based host cell lysis provides effective host DNA depletion in blood samples spiked with clinically relevant sepsis pathogens, including *E. coli*, *S. aureus*, *K. pneumoniae*, and *E. faecalis* (Moragues-Solanas et al., 2024). However, they have not evaluated various enzyme concentrations to determine their effects on host DNA depletion and subsequent bacterial DNA extraction. In our previous study, we tested the host DNA depletion capability of HL-SAN and M-SAN using 250 U (10 μL) in clinical urine samples (Bellankimath et al., 2025). The results showed effective depletion of host DNA, which is enough for accurately identifying bacterial pathogens and antibiotic resistance genes in clinical urinary tract infection samples using nanopore sequencing.

In this study, we demonstrated that using both enzyme concentrations (250 U and 500 U) effectively depleted host DNA in *E. coli* and *S. aureus* blood cultures. When comparing the tested concentrations among *S. aureus* samples, slightly better host DNA depletion was observed with 500 U (20 μL) of both enzymes compared to their respective 250 U (10 μL). However, this was not observed in *E. coli* samples, as both the tested concentrations of HL-SAN and M-SAN achieved similar host DNA depletion. When comparing the enzymes in *E. coli* samples, M-SAN showed less host DNA depletion but greater bacterial DNA recovery. In contrast, HL-SAN exhibited enhanced depletion, accompanied by a corresponding loss of bacterial DNA. This host depletion trend was also observed among *S. aureus* samples. However, the bacterial DNA recovery between HL-SAN and M-SAN showed minimal differences.

Therefore, it can be concluded that although changing enzyme concentrations has a slight effect on host DNA depletion in blood cultures, the overall host depletion at lower concentrations remains sufficient for downstream processes. Using a lower enzyme concentration would halve the cost per sample, since only half the units would be used. Generally, in cases of infections such as BSIs or sepsis, the host’s immune response is heightened, leading to the release of large numbers of leukocytes into the bloodstream (Liang and Yu, 2022). Therefore, the amount of host DNA released from leukocytes in sepsis samples would be higher than in spiked healthy samples, where no immune response is triggered. However, upon preliminary testing with positive clinical blood cultures, 250 U (10 μL) of HL-SAN provided sufficient host DNA depletion, as shown by qPCR results.

### Adjusting salt levels to influence enzyme activity has little impact on host DNA depletion

This study used different salt concentrations as buffers for HL-SAN and M-SAN to examine their impact on enzyme activity and overall host DNA depletion in blood cultures. Results indicated that the buffer with higher salt concentrations (2.5 M NaCl and 50 mM MgCl_2_) achieved slightly higher host DNA depletion than buffers with lower salt concentrations. However, the differences in host DNA depletion were not statistically significant when altering the concentrations of NaCl and MgCl_2_ in the buffers. HL-SAN and M-SAN are salt-active nucleases and can withstand high salt levels. The manufacturer (ArcticZymes) recommends a concentration range of 125 mM – 250 mM NaCl and 4–15 mM MgCl_2_ for M-SAN and 400–700 mM NaCl and 5–50 mM MgCl_2_ for optimal HL-SAN activity. However, the optimal activity across different sample types, such as blood, urine, and respiratory samples, can vary due to the presence of salt or the salt’s absorption by the sample matrix in the buffer.

Endonucleases catalyze the cleavage of phosphodiester bonds within the DNA double helix. They introduce single- or double-stranded breaks through hydrolysis of the phosphodiester linkage, specifically targeting the P–O3′ bond, and produce DNA fragments with 5′-phosphate and 3′-hydroxyl termini (Guéroult et al., 2010). Along with the HL-SAN and M-SAN that we tested in this study, numerous nucleases such as Benzonase (Sigma Aldrich Merck), DNase I, Denarase (c-Lecta), and DNaseMe (Qiagen) are commercially available (Kawka et al., 2021; von Elling-Tammen et al., 2025). Most of the nucleases require MgCl_2_ or CaCl_2_ as a cofactor for their activity.

Von Elling-Tammen *et al*. tested different salt concentrations to compare the activity of Benzonase, Denarase, Saltonase, MNase, DnaseMe, M-SAN, and SAN (HL-SAN) (von Elling-Tammen et al., 2025). These nucleases were tested under salt conditions of 2 mM MgCl_2_ and higher concentrations recommended by the respective manufacturers. The results showed that Benzonase, Denarase, MNase, and M-SAN demonstrated efficient DNA digestion at 2 mM or higher MgCl₂ and CaCl₂ concentrations, with MNase requiring CaCl₂ as a cofactor (von Elling-Tammen et al., 2025). Benzonase and Denarase have been shown to perform optimally at lower NaCl concentrations, while M-SAN performed exceptionally well even with higher concentrations of salt. SAN enzymes are structurally engineered to function in high salt environments due to their intramolecular interactions that are less affected by ionic strengths. These results align with our observations in this study, showing that using 15 mM or 50 mM MgCl₂ and varying NaCl concentrations had no significant effect on the enzymatic activity of HL-SAN and M-SAN. Additionally, in our previous study, we demonstrated that HL-SAN and M-SAN effectively removed host DNA from clinical urine samples using higher salt concentrations of 2.5 M NaCl and 50 mM MgCl_2_ (Bellankimath et al., 2025).

### Reducing bead-beating time improved the recovery of larger DNA fragments

Nanopore rapid barcoding protocols suggest a starting DNA fragment length of greater than 30 kb, aiming to generate read lengths of 8 kb (Heavens et al., 2021). While high molecular weight DNA input is recommended, ultra-long DNA fragments may be counterproductive for library preparation, as they may be lost during the process due to inefficient binding to or elution from the solid phase reversible immobilization (SPRI) beads. Although fragmentation is not necessary for nanopore sequencing, it can assist in creating uniform libraries from samples with high molecular weight DNA or low input DNA (Oxford Nanopore Technologies, 2021). Therefore, it is crucial to optimize the DNA extraction process for isolating high-quality and high-weight DNA to achieve high throughput using nanopore sequencing (Gand et al., 2023). To achieve this, typically in biological fluids, host DNA is depleted using saponin and nucleases, and then microbial DNA is extracted. In addition to other methods, mechanical lysis with various types of beads is commonly used to break open microbial cells and release their DNA (Shehadul Islam et al., 2017; Frau et al., 2019). BB is critical for gram-positive bacteria because their thick cell walls are difficult to break down with chemical methods (Li et al., 2020). Although BB can enhance nucleic acid yield, too much BB can cause DNA shearing, which affects subsequent processes.

In this study, we used the BiOstic kit, which employs BB to lyse the cells. The default kit protocol recommends using 10 min of continuous BB. However, we observed that using the 10-min BB method caused DNA shearing, which reduced sequencing throughput, especially for gram-negative bacteria such as *E. coli*. Therefore, the BB time was shortened to three cycles, each consisting of 2 minutes of BB followed by 1 minute of incubation on ice. The average read lengths significantly increased in both *E. coli* and *S. aureus* when 6 min of BB was used, compared to 10 min. Similarly, the number of nanopore sequencing reads mapping to the target bacteria significantly increased, while host reads decreased, following 6 min of BB compared to 10 min. BB for 10 min provided higher DNA yields at all tested conditions, except in the *E. coli* sample processed with M-SAN, where the DNA yield was higher with 6 min of BB (300 ng) compared to 10 min of BB (105 ng). The reason for this might be excessive shearing of *E. coli* DNA during 10 min of BB, followed by its loss during the washing steps. The BiOstic kit is designed for DNA extraction directly from blood cultures and does not include any host DNA depletion mechanisms. In this study, the blood culture samples used for extraction were host DNA-depleted with nucleases, resulting in fewer host cells. Therefore, a higher beads-to-cells ratio might have caused fragmentation of the microbial DNA due to extended BB.

Previously, BB times of 0, 3, 10, 15, and 20 min have been tested on bacterial and fungal communities, and the results showed that 20 min of BB is the most effective for recovering most of the microbial DNA (Frau et al., 2019). The recovery of high-integrity DNA using BB also depends heavily on the instrument type, speed, and bead type and quantity. Similar to earlier studies, we observed that increasing the BB time decreased the recovery of high-quality DNA from the gram-negative bacterium *E. coli* (Zhang et al., 2021). Therefore, we recommend a BB time of 6 min to balance effective DNA recovery from both gram-positive and gram-negative bacteria.

### Clinical blood culture testing provided efficient host depletion and bacterial DNA recovery

The optimized protocol was validated on clinical blood cultures, which were positive for the most common bacterial pathogens causing bloodstream infections. The optimized method demonstrated efficient performance in DNA extraction from positive clinical blood cultures. Several previously published studies have used host DNA depletion strategies for positive blood culture samples (Anson et al., 2018; Harris et al., 2024). Anson et al. (2018) used a differential centrifugation, filtration, and selective lysis-based method to deplete the host cells, followed by DNA extraction with commercially available kits. They found that the BiOstic kit yielded the highest mean DNA concentration. In our study, we also observed higher DNA yield with the BiOstic kit, even after host DNA depletion, although we did not directly compare it with any commercial kits. Similarly, Harris et al. (2024) used MolYsis Complete and Basic kit for host depletion and later extracted the DNA with either the Mini-Pure extraction or UltraClean extraction method. They showed that the UltraClean method is optimal for host-depleted and microbial-enriched DNA that is compatible with the downstream molecular applications. However, the disadvantage of this method is the cost, as two commercially available kits must be used for host depletion and subsequent microbial DNA extraction. However, in our method, we have optimized host DNA depletion, while DNA extraction is performed using a commercial kit.

## Conclusion

In summary, these results demonstrate that a 4% saponin solution, followed by digestion with HL-SAN at 250 U (10 μL), showed efficient host depletion in spiked and clinical blood cultures from patients. Additionally, different concentrations of NaCl and MgCl_2_ used as enzyme buffers did not significantly affect enzyme activity or host DNA depletion. The shorter BB time of 6 min is recommended because it yields highly intact DNA and longer fragments compared to the standard 10-min BB from the BiOstic kit. Furthermore, the optimized method in this study is compatible with downstream applications such as qPCR and nanopore sequencing. The findings provide valuable information for host DNA depletion using saponin and SAN enzymes. The protocol was refined using the two most common sepsis-causing pathogens, *E. coli* and *S. aureus*, and then validated with additional relevant pathogens and clinical positive blood cultures. In this study, host DNA depletion was optimized and validated specifically with selective aerobic sepsis-causing bacteria. Future research should examine the effectiveness of this method on other anaerobic sepsis-related pathogens and in samples with lower bacterial loads. Additionally, in our future studies, the method will be further optimized and tested on a larger cohort of clinical blood cultures.

## Data Availability

The dataset presented in this study is available online at NCBI Sequence Read Archive with accession number PRJNA1302749.
