# Supplementary material for: Development and optimization of host DNA depletion in blood cultures using a saponin and salt-activated nuclease-based method
Source: Front Microbiol. 2026 Apr 8;17:1784408. doi: 10.3389/fmicb.2026.1784408 (PMC13099825; doi:10.3389/fmicb.2026.1784408)
Supplement: Supplementary file 2 [file Supplementary_file_1.pdf]

Development and optimization of host DNA depletion in blood cultures using a saponin and salt-activated nuclease-based method

Jawad Ali[1], Anurag Basavaraj Bellankimath[1], Jonathan Hira[1], Crystal Chapagain[1], Silje Therese Opgård[3], Gunnar Skov Simonsen[3,4], Rafi Ahmad[1,2*]

[1] Department of Biotechnology, University of Inland Norway, Holsetgata 22, 2317, Hamar, Norway

[2] Institute of Clinical Medicine, Faculty of Health Sciences, UiT - The Arctic University of Norway, Hansine Hansens veg 18, 9019, Tromsø, Norway

[3] Department of Microbiology and Infection Control, University Hospital of North Norway, Tromsø, Norway, Hansine Hansens veg 67, 9019 Tromsø, Norway

[4] Department of Medical Biology, Faculty of Health Sciences, UiT – The Arctic University of Norway, Hansine Hansens veg 18, 9019, Tromsø, Norway

Email: jawad.ali@inn.no, anurag.bellankimath@inn.no, jonathan.hira@inn.no, crystal.chapagain@inn.no, Silje.Therese.Opgard@unn.no, Gunnar.Skov.Simonsen@unn.no rafi.ahmad@inn.no*

Supplementary Information

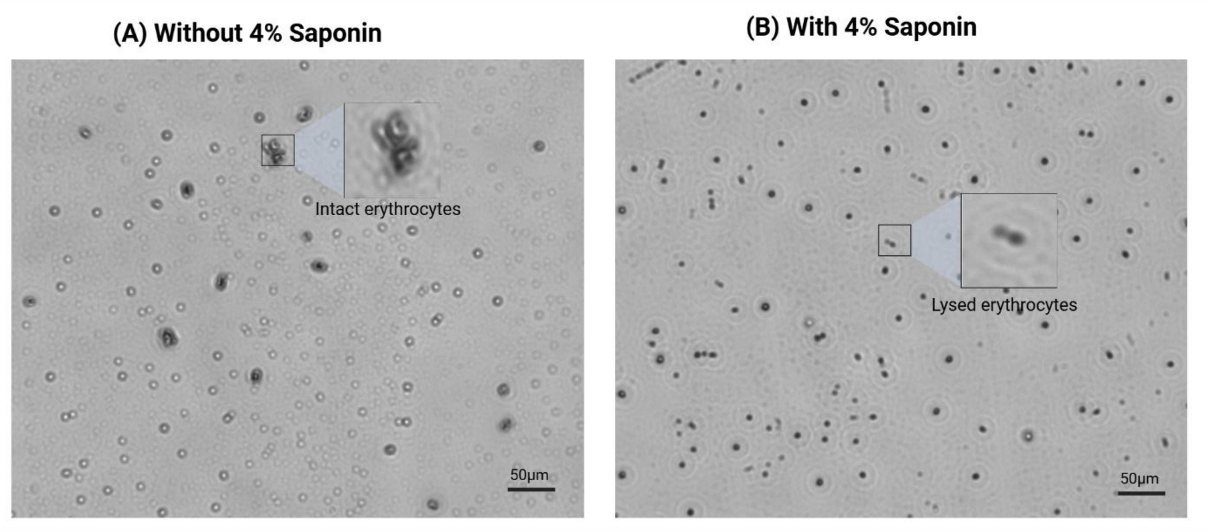

Supplementary Figure 1. Illustrates the targeted cellular lytic effect of saponin at 4% concentration. **(A)** Without saponin, healthy intact erythrocytes are observed, but **(B)** lysed erythrocytes are prominent in the presence of saponin.

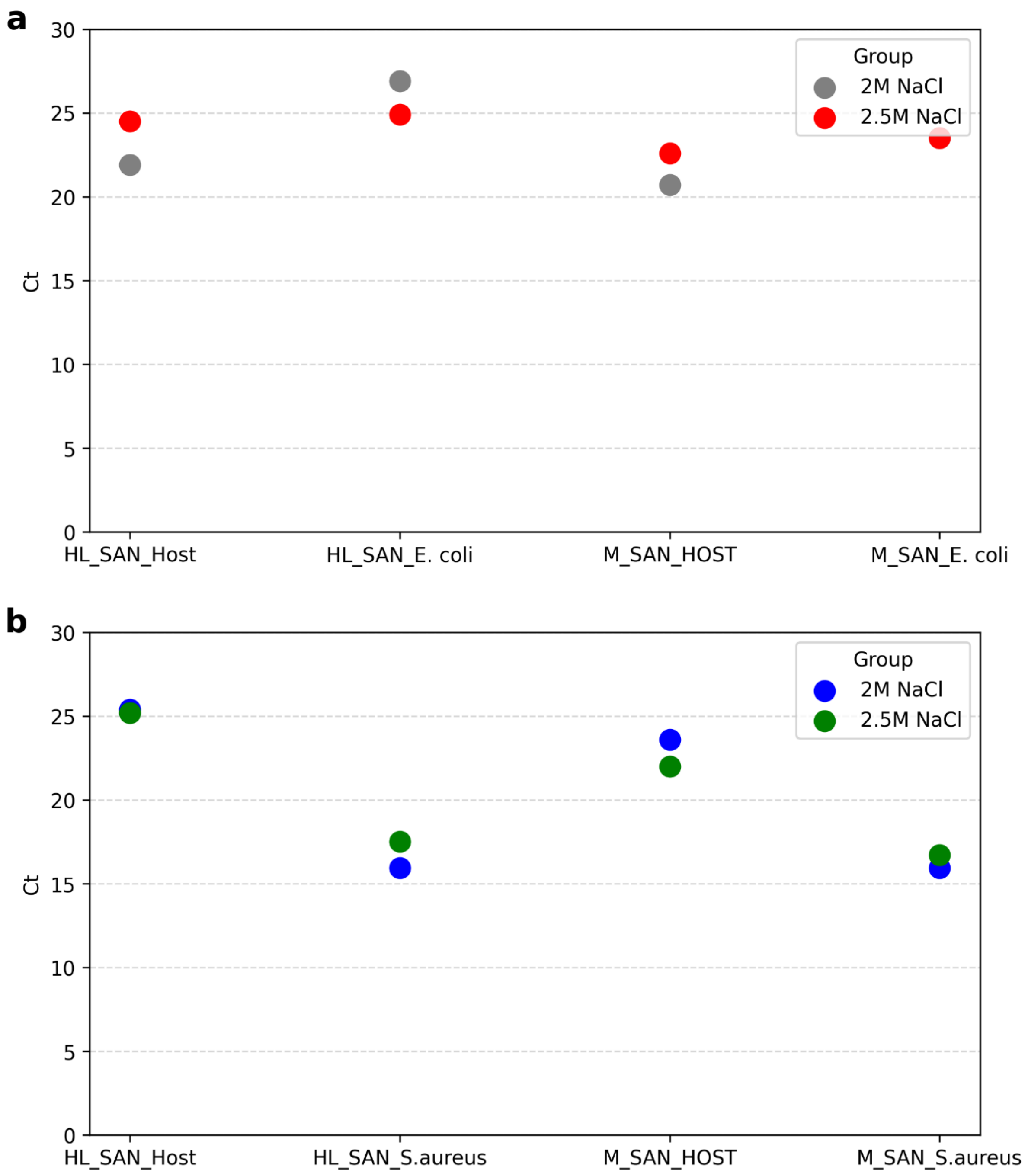

Supplementary Figure 2. The effect of NaCl concentrations on HL-SAN and M-SAN activity for host DNA depletion. The concentration of HL-SAN and M-SAN used are 250 U. The average Ct values are presented for both *E. coli* and *S. aureus* and as well as the host primers. The concentration of $MgCl_2$ is maintained at a constant 50 mM **(a)** *Escherichia coli* and **(b)** *Staphylococcus aureus*. 2M/2.5M = NaCl.

Supplementary Table 1. Information about the primers used for the qPCR amplification of host and bacterial DNA from blood cultures.

**Target species**	**Target gene**	**Amplicon size (bp)**	**Primer sequences**	**References**
Sheep	*GAPDH*	130	FP: GTCCGTTGTGGATCTGACCT<br>RP: GGAGACAACCTGGTCCTCAG	[1]
E. coli	*UspA*	850	FP: CCGATACGCTGCCAATCAGT<br>RP: ACGCAGACCGTAGGCCAGAT	[2]
S. aureus	*Nuc*	65	FP: GGGTTGATACGCCAGAAACG<br>RP: TGATGCTTCTTTGCCAAATGG	[3]
K. pneumoniae	*Khe*	428	FP: TGATTGCATTCGCCACTGG<br>RP: GGTCAACCCAACGATCCTG	[4]
P. aeruginosa	*phzA2*	325	FP: GTTTACCGACAACCTGGAA<br>RP: GCAATAGCCCTGCGGATAC	[5]
E. faecium, E. faecalis	*GroEs*	185	FP: GGGTTGATACGCCAGAAACG<br>RP: TGATGCTTCTTTGCCAAATGG	[6]

Supplementary Table 2. Host DNA depletion using different concentrations of HL-SAN and M-SAN, followed by extraction with the BiOstic kit. HL-10/20 = 10/20 µL (250/500 U) HL-SAN, M-10/20 = 10/20 µL (250/500 U) M-SAN.

**Samples**	**Endonuclease used**	**Enzyme units (U)**	**DNA yield (ng)**	**Nanodrop quality check**		**Average size (bp) TapeStation**	**DIN value**	**Average CT (Bacteria)**	**Average CT (Host)**
				**260/280**	**260/230**				
E. coli	HL-SAN	250	55	1.4	0.3	4007	3.8	24.9	24.5
	HL-SAN	500	45	1.4	0.3	2809	3.1	25.5	24.9
	M-SAN	250	105	1.5	0.2	9792	7	23.5	22.6
	M-SAN	500	90	1.5	0.5	6874	6.6	23.6	22.8
	No depletion (Biostic kit only)	n/a	1000	1.7	0.9	7321	6.4	21.3	19.3
S. aureus	HL-SAN	250	75	1.3	0.1	8638	6.2	17.5	25.2
	HL-SAN	500	115	1.3	0.5	13632	6.6	16.6	26.7
	M-SAN	250	230	1.6	1.1	12536	7.3	16.7	22
	M-SAN	500	145	1.6	0.3	14575	7.5	17.1	23
	No depletion (Biostic kit only)	n/a	800	1.7	1.7	N/A	N/A	16.4	20

Supplementary Table 3. Host depletion and DNA extraction from blood cultures using 2.5 M NaCl (constant) and 15 and 50 mM $MgCl_2$ as buffer for enzyme activity.

Samples	Endonuclease Used (250 U)	Biological replicates	$MgCl_2$ conc (mM)	DNA yield (ng)	Average CT (Bacteria)	Average CT (Host)
E. coli	HL-SAN	1	15	172	19.3	22.9
		2	15	145	19.6	23.1
		1	50	157	19.5	22.9
		2	50	111	19.6	23.6
	M-SAN	1	15	176	19.1	22.1
		2	15	207	18.6	21.7
		1	50	330	18.3	20.8
		2	50	286	18.6	21.3
S. aureus	HL-SAN	1	15	198	19.7	22.5
		2	15	235	20.2	22.2
		1	50	307	19.4	22.2
		2	50	175	19.7	23.3
	M-SAN	1	15	268	20.2	21.3
		2	15	177	20.7	22
		1	50	358	19.8	20.8
		2	50	168	20.1	21.6

Supplementary Table 4. Host depletion and DNA extraction from blood cultures using 0.5 M NaCl and 15 mM $MgCl_2$ as buffer for enzyme activity.

Samples	Endonuclease used (250 U)	NaCl conc (M)	$MgCl_2$ conc (mM)	Biological replicates	DNA yield (ng)	Average CT (Bacteria)	Average CT (Host)
E. coli	HL-SAN	0.5	15	1	194	19.3	22.4
				2	255	19.3	21.6
		2.5	50	1	157	19.5	22.9
				2	111	19.6	23.6
	M-SAN	0.5	15	1	309	19.5	21.5
				2	295	19.7	21.4
		2.5	50	1	330	18.3	20.8
				2	286	18.6	21.3
S. aureus	HL-SAN	0.5	15	1	207	20.2	22.3
				2	183	20.1	22.5
		2.5	50	1	307	19.4	22.2
				2	175	19.7	23.3
	M-SAN	0.5	15	1	395	19.7	20.6
				2	486	19.8	20.7
		2.5	50	1	358	19.8	20.8
				2	168	20.1	21.6

Supplementary Table 6. Testing of the final optimized method on clinical blood culture samples from patients.

Samples (Blood cultures)	Endonuclease used	Enzyme units (U)	DNA yield (ng)	Average CT (Bacteria)	Average CT (Host)
E. coli	HL-SAN	250	11550	16.6	32.7
S. aureus	HL-SAN	250	4740	16.9	40.0
K. pneumoniae	HL-SAN	250	17040	14.9	40.0
E. faecium	HL-SAN	250	13440	36.0	40.0
P. aeruginosa	HL-SAN	250	168	18.8	30.4

Supplementary Table 7. Testing of the final optimized method on spiked culture with sepsis relevant bacterial pathogens using 4% saponin, 250 U HL-SAN and a buffer consisting of 2.5 M NaCl and 50 mM $MgCl_2$.

**Samples**	**Endonuclease used**	**Enzyme units (U)**	**DNA yield (ng)**	**Average CT (Bacteria)**
K. pneumoniae	HL-SAN	250	55.5	19.6
E. faecalis	HL-SAN	250	12.6	24.6
E. faecium	HL-SAN	250	61.8	18.2
P. aeruginosa	HL-SAN	250	32.4	22.4

References

1. Gebreselassie, G. *et al.* Correction: Genomic mapping identifies two genetic variants in the MC1R gene for coat colour variation in Chinese Tan sheep. *PLOS ONE* **16**, e0245674 (2021).
2. Anastasi, E. M. *et al.* Prevalence and Persistence of Escherichia coli Strains with Uropathogenic Virulence Characteristics in Sewage Treatment Plants. *Applied and Environmental Microbiology* **76**, 5882–5886 (2010).
3. Ahmadi, A., Khezri, A., Nørstebø, H. & Ahmad, R. A culture-, amplification-independent, and rapid method for identification of pathogens and antibiotic resistance profile in bovine mastitis milk. *Front Microbiol* **13**, 1104701 (2022).
4. Sun, Y., Wu, H. & Shen, D. Detection and Analysis of Regional Trends of Klebsiella pneumonia Causing Liver Abscess. *Clinical Microbiology: Open Access* **4**, 1–5.
5. Wang, C. *et al.* Pseudomonas aeruginosa Detection Using Conventional PCR and Quantitative Real-Time PCR Based on Species-Specific Novel Gene Targets Identified by Pangenome Analysis. *Front Microbiol* **13**, 820431 (2022).
6. Teng, L.-J. *et al.* Determination of Enterococcus faecalis groESL Full-Length Sequence and Application for Species Identification. *J Clin Microbiol* **39**, 3326–3331 (2001).
